# The Association of Smartphone Usage Duration with Physical Fitness among Chinese University Students

**DOI:** 10.3390/ijerph19010572

**Published:** 2022-01-05

**Authors:** Wang Li, Yufei Cui, Qiang Gong, Cong Huang, Feng Guo

**Affiliations:** 1 Department of Physical Education, Institute of Exercise Epidemiology, Huaiyin Institute of Technology, Huaian 223003, China; kakuhoulove@hotmail.com; 2 Department of Medicine and Science in Sports and Exercise, Graduate School of Medicine, Tohoku University, Sendai 9808575, Japan; qianggong0922@yahoo.co.jp; 3 Department of Physical Education, Zhejiang University, Hangzhou 310007, China; cohuang@zju.edu.cn

**Keywords:** smartphone use, physical fitness, university students, cross-sectional study

## Abstract

Background: The use of smartphones has become increasingly prevalent in recent years, especially among the youth. However, smartphone overuse has been reported to be related to several negative mental and physical health outcomes. Although the association between smartphone use and physical fitness has been investigated in several studies, these studies only focused on specific elements of physical fitness, such as grip strength. In addition, evidence on young adults is limited. Thus, this study aimed to examine the association between the duration of smartphone use and physical fitness among Chinese university students. Methods: A total of 11,242 university students volunteered to participate in the study. The duration of smartphone use was assessed using a self-reported questionnaire. Physical fitness tests consisted of a 50-m sprint and vital capacity tests for both sexes, a 1000-m run and pull-up test for male students, and an 800-m run and sit-up test for female students. Results: The duration of smartphone use among the participants was 5.4 h/day for male students and 6.1 h/day for female students on average. After adjusting for confounding factors, in male students, a long duration of smartphone use was significantly associated with a slow 50 m sprint and 1000 m run time, lower pull-up times, and poor vital capacity (*p* = 0.004, 0.002, 0.002 and 0.040, respectively). In female students, a long duration of smartphone use was associated with a slow 800 m run time (*p* < 0.001). Conclusion: This study found that longer duration of smartphone use was associated with lower physical fitness among Chinese university students. The duration of smartphone use may be an influencing factor for physical fitness.

## 1. Introduction

Smartphones have evolved greatly from cellphones in the past decade and have had an obvious impact on people’s lives. Today’s smartphones can not only make phone calls, but also easily access the Internet, play games, take photos, listen to music, and shop. These functions bring people convenience, and smartphone usage has increased dramatically over the years. However, as smartphones usage increases, the likelihood of problematic smartphone use also increases, which may lead to negative health outcomes. Some studies have indicated that smartphone overuse is associated with depressive symptoms, memory and concentration problems, changes in eating behaviors, and poor sleep quality [1,2,3,4].

Young adulthood is a period of life in which people aged 18–24 years have passed through adolescence, but have not yet fully assumed the role of an adult [5]. In China, most young adults at this age are university students. Their lifestyles change during this period because they leave their families and begin to live alone. They manage their own studies and lives. Lifestyles formed during this period may influence their whole life. However, university students have a higher frequency of smartphone use in China, owing to its use for online classes and online homework. Moreover, they have more free time than in high school and are no longer under their parents’ discipline; it may be difficult to control their smartphone overuse. Therefore, maintaining appropriate smartphone use may be important for university students.

In addition to its effect on mental health, smartphone overuse is reported to be related to poor physical health. Studies have demonstrated that long durations of smartphone use is associated with fatigue [6], headaches, and neck pain [7,8]. As poor mental and physical status are risk factors for physical inactivity, it could be considered that smartphone use may also lead to lowered physical activity, and may consequently cause physical fitness decline. Physical fitness is considered as one of the most important health markers [9]. Previous studies showed that some physical fitness indicators are directly related to improvements in cardiovascular health [10], and are inversely associated with the risk of type 2 diabetes [11], hypertension [12], and all-cause mortality [13]. However, to our knowledge, few studies examined the association between smartphone usage and physical fitness, including a Spanish study of 501 adolescents, aged 12–18 [14]; a Korean study of 237 university students [15]; and an American study of 305 college students [16]. All these studies examined the association between mobile phone use and physical fitness, and revealed that high usage of mobile phones was related to poor physical fitness. However, some of these studies only focused on specific elements of physical fitness, while others only focused on high school students. In addition, no studies have specifically provided evidence on the relationship between the duration of smartphone use and physical fitness in Chinese university students, who lead a different lifestyle from students in other countries. In consideration of these differences, it is relevant to investigate this association in Chinese university students. Therefore, we designed a cross-sectional study to examine whether the duration of smartphone use is associated with physical fitness among Chinese university students.

## 2. Materials and Methods

### 2.1. Participants

This cross-sectional study was conducted at the Huaiyin Institute of Technology, a university in Huaian City, Jiangsu, China, and was based on an annual physical health examination for university students in 2018. The Ethics Committee of the Huaiyin Institute of Technology approved the study. A questionnaire survey was conducted before the examination, and all students were invited to participate; they did so voluntarily. After providing information about the study, written informed consent was obtained from all study participants before the survey. In total, 12,580 students agreed to participate in the study and provided their consent for data analysis. Participants with missing data on their smartphone use (*n* = 533) and physical fitness (*n* = 1) were excluded. Furthermore, 804 students who had missing data on body mass index, race, depressive symptoms, sleep, living status, living expenses, physical activity, smoking, and drinking status were also excluded. Thus, the final study size was 11,242 (6770 male and 4472 female students).

### 2.2. Assessment of Smartphone Use

Duration of smartphone use was assessed by the question, “How long do you usually use your smartphone in a day?”. If students did not know the exact duration of smartphone use, they would be asked to check the screen time on the smartphone (which is an option available for all smartphones). The total duration of smartphone use was calculated from the students’ responses.

### 2.3. Assessment of Physical Fitness

All measurements of physical fitness were based on the National Student Physical Health Standard (revised in 2014) [17], which was developed for monitoring and evaluating the physical fitness changes of Chinese students (from primary school to university). Some contents in measurement were different between men and women since it was developed; for example, a 1000 m run is used to evaluate middle distance running ability for men, and an 800 m run is for women. The pull-up test is used to evaluate upper body strength in men and the sit-up test is used for women. We conducted the following physical fitness tests by using the criteria for university students:

#### 2.3.1. 50 m Sprint

After a warm-up, participants performed a 50 m spring on a standard track surface, with the time recorded. They were encouraged not to slow down before crossing the finish line. The running time was recorded in seconds.

#### 2.3.2. 1000 m and 800 m Run

The running tests were performed on a standard track surface: a 1000 m run for males, and an 800 m run for females. The total time taken to complete the distance was recorded in minutes and seconds.

### 2.4. Pull-Ups

Participants grasped the overhead bar using an overhand grip (palms facing away from the body) with the arms fully extended. The participants then raised their body until the chin cleared the top of the bar, and then lowered it to a position with the arms fully extended. The pull-ups were to be performed in a smooth motion. Jerky motions, swinging the body, and kicking or bending the legs were not permitted. As many complete pull-ups as possible were performed.

### 2.5. Sit-Ups

Participants were told to lie on a cushioned floor with knees bent at approximately right angles, with their feet flat on the ground. Their hands were placed behind their heads. The feet were anchored by a partner. Then, after a start instruction, participants squeezed their stomach and let their back raise high enough for their elbow to touch the tops of their knees, then return to the starting position. As many complete sit-ups as possible were performed.

### 2.6. Vital Capacity

Vital capacity was measured using a spirometer (JH-1663, Jihao Electron Co., Changzhou, China), with the participants in a standing position. Participants were instructed to perform maximal slow expiration, starting from total lung capacity, through a mouthpiece connected to the spirometer. Results are shown as vital capacity (mL)/body weight (kg).

### 2.7. Assessment of Other Variables

Body weight and height were measured before physical measurements. Body mass index (BMI) was calculated as weight (kg)/height (m)^2^. Daily physical activity (PA) was estimated using the International Physical Activity Questionnaire (IPAQ) [18]. The total daily PA (metabolic equivalents [METs] × h/week) was calculated. PA was categorized into tertiles (low, medium, and high). Depressive symptoms were determined using the Zung Self-Rating Depression Scale (SDS) [19]. An SDS score ≥ 45 was considered the cutoff point indicating relatively mild or severe depressive symptoms [20]. Sex, grade, living expenses, race, living status, sleep duration, smoking, and alcohol drinking status were assessed using a self-reported questionnaire. Grade was categorized into first-year students, sophomores, and juniors. Living expenses were divided into three categories: low (≤1000 yuan/month), medium (1001–1500 yuan/month), and high (>1500 yuan/month). Race was categorized into Han and minority races. Living status was categorized as dormitory or other. Sleep duration was divided into 7–8 h of sleep duration and other sleep durations. Smoking status was categorized as smoker and non-smoker groups. Drinking status was categorized as non-drinker, drinking 1–2 times/week, and drinking >2 times/week.

### 2.8. Statistical Analysis

Statistical analysis was performed using the Statistical Package for Social Science (SPSS) (version 24.0; IBM Corporation, Armonk, NY, USA) for Windows. The differences in variables among the duration of smartphone use categories in participant characteristics were examined by *t*-test or analysis of variance (ANOVA) for continuous variables or by the χ^2^ test for categorical variables. Multivariate linear regression analysis was used to examine the adjusted associations between the duration of smartphone use and physical fitness. The duration of smartphone use was used as an independent variable. Physical fitness was used as a dependent variable. Grade, BMI, race, living expenses, PA, living status, smoking and drinking status, depressive symptoms, and sleep duration were considered as confounding factors. The results are presented as the betas with 95% confidence intervals (95% CIs), and the level of significance was set at *p* < 0.05. Considering that differences in physical fitness and smartphone use exist between men and women, all analyses were performed separately by sex.

## 3. Results

### 3.1. Sample Characteristics

The characteristics of 11,242 participants (60.2% men and 39.8% women) according to sex are presented in Table 1. Compared to female participants, male participants had higher values for BMI, vital capacity, and lower values for duration of smartphone use and 50 m sprint time. The proportion of senior students, low living expenses, drinking 1–2 times per week, and drinking >2 times per week were higher in male participants than in female participants. The proportion of first-year students, sophomores, juniors, minority races, students with medium and high living expenses, nonsmokers, nondrinkers, and students obtaining 7–8 h sleep duration per night were higher in female than in male participants.

The correlations between each physical fitness component are presented in Table 2. In male participants, the 1000 m run was significantly positively associated with the 50 m run and vital capacity. Pull-up was inversely associated with the duration of smartphone use, 50 m sprint, and 1000 m run. Vital capacity is inversely associated with 50 m sprint and pull-up. In female participants, 800 m run was positively associated with the duration of smartphone use and 50 m sprint. Sit-up is inversely associated with 50 m sprint and 800 m run. Vital capacity was inversely associated with 50 m sprint and positively associated with sit-up.

### 3.2. Smartphone Uses and Physical Fitness in Males

Table 3 shows adjusted association between duration of smartphone use and physical fitness in male participants. In the crude model, results show that the duration of smartphone use is only inversely associated with pull-up. In the adjusted model, the duration of smartphone use is positively associated with 50 m sprint and 1000 m run and inversely associated with pull-up and vital capacity.

### 3.3. Smartphone Uses and Physical Fitness in Females

Table 4 shows the adjusted association between duration of smartphone use and physical fitness in females. A significantly positive association was found between the duration of smartphone use and the 800 m run in both crude and adjusted models. However, no significant association was found with other physical fitness in female participants.

## 4. Discussion

In this study, we evaluated the relationship between the duration of smartphone use and physical fitness in a sample of Chinese university students. The main findings of this study were that long durations of smartphone use are associated with longer 50 m sprint times, longer 1000 m run times, and a smaller number of pull-ups in male students; it is also associated with longer 800 m run time in female students. These associations did not change after adjusting for a number of potentially confounding variables. To our knowledge, no previous study has investigated the relationship between the duration of smartphone use and physical fitness in the Chinese young adult population. Thus, our study could provide important information on health education as well as evidence for future research in the field of smartphone usage and physical fitness.

Smartphone use duration averaged 7.8 h in an Arabian study aged 18–30 years [21]. Another Arabian study showed 6.7 h of smartphone use duration in 2367 university students [22]; in a Canadian study of 104 university students, smartphone use duration was 5.1 h [23]. The duration of smartphone use among university students in most studies was 5–8 h; the 5.8 h using duration (average of males and females) found in our study is also in this range. Several studies have reported higher rates of smartphone use and lower muscle strength. A study explored the association between smartphone usage duration and hand grip and pinch grip strength among young people aged 19–23 years. They found a weak but significant inverse association between the variables [21]. Another observational study indicated that high levels of smartphone use was associated with diminished grip strength in 60 children aged between 9 and 15 years [24]. Conversely, an interventional study compared grip strength between two groups (<4 h of smartphone use, and ≥4 h of smartphone use) based on the duration of smartphone usage. The results showed no difference in grip strength between the two groups [25]. All these previous studies investigated the relationship between smartphone usage and grip strength. However, their results were inconsistent; additionally, they only evaluated upper body strength, and other physical fitness markers, such as lower body strength and endurance, were not considered. Meanwhile, a Spanish study described the association between the use of mobile phones and physical fitness in high school students. Within the boys’ group in this study, physical fitness performance was higher in the low mobile phone use group than in the high mobile phone use group for the abdominals test, the medicine-ball throw, the broad jump, and the trunk-flexion test. However, for the girls’ group, no differences among the mobile phone use groups were found [14]. A study on 237 Korean university students showed that longer smartphone use was related to weaker grip strength and poorer performance in sit-ups, side steps, and Sargent jumps [15]. The results of these studies are partially consistent with the current study. We examined the association between mobile phone use and physical fitness in young population, and concluded that higher mobile phone use was associated with lower physical fitness performance. However, the participants in our study were from a different country, representative of a different group, and included different physical fitness assessments. Thus, our study strengthened the evidence on the association between mobile phone use and physical fitness, and expanded this association to Chinese university students.

The mechanism for the associations shown in this study is currently unknown. There are some possible explanations for the association between the duration of smartphone use and physical fitness. A previous study on adults indicated that 18.8% of smartphone users experienced symptoms related to musculoskeletal disorders [26]. In addition, fatigue and pain were proven to occur more easily with the use of touch-screen computers and smartphones [6,27]. These factors may directly cause physical fitness performance decline. Meanwhile, long duration of smartphone use could not only take time away from more physically active behaviors and decrease the metabolic rate, but is also related to increased body fat mass [28,29]. These factors may indirectly cause a decrease in physical fitness. Furthermore, long smartphone use at night may lead to late bedtimes, causing poor sleep quality or leading to waking up late in the morning and skipping breakfast. Poor sleep quality and low frequency of breakfasts are risk factors for low physical fitness; therefore, long duration of smartphone use may be associated with poor physical fitness.

Our study had several limitations. First, because of the cross-sectional design of our study, causality could not be established. Thus, a longitudinal study is needed to determine the relationship between the duration of smartphone use and physical fitness. Second, as all participants were Chinese university students, our findings cannot be broadly applied to other ages or ethnicities. Third, this study was conducted with 30 instructors. Although every instructor was trained before the physical measurement, there may be measurement errors between them. Fourth, the use of self-reported data on some assessments could result in underreporting or unintentional recall bias. Fifth, we only assessed the duration of smartphone use by a single question in this study. We could not use standardized scales to multi-evaluate smartphone use due to the limited survey time. Finally, we used some confounding factors to adjust for the association between duration of smartphone use and physical fitness. We cannot exclude other unknown factors that affect the association between smartphone use and physical fitness.

## 5. Conclusions

This study examined the relationship between the duration of smartphone use and physical fitness among Chinese university students. Our results could be important from a public health point of view, as the results demonstrated that a longer duration of smartphone use was associated with a lower level of physical fitness in university students. Students with long duration of smartphone use should be mindful of the potential risks of physical fitness. Moreover, these findings suggest to educators, students, and their parents that students could develop positive behaviors and lifestyles by reducing the use time of smartphones, so as to reduce or eliminate the risk factors affecting physical health. Further prospective or randomized studies should evaluate the causal relationship between smartphone use and each element of physical fitness.

## Figures and Tables

**Table 1:** Basic characteristics of male and female participants.

	Male	Female	*p* Value ^a^
*n* (%)	6770 (60.2)	4472 (39.8)	
BMI (kg/m^2^) ^b^	22.0 (22.0, 22.1) ^c^	20.4 (20.3, 20.5)	<0.001
Grade (%)			
Freshman	29	31.3	0.011
Sophomore	28.8	31.6	0.002
Junior	26.3	28.6	0.007
Senior	15.9	8.5	<0.001
Minority race (%)	3.9	5.7	<0.001
Living expenses (%)			
Low	39.7	35	<0.001
Medium	51	53.8	0.004
High	9.3	11.2	0.001
Living status (dormitory; %)	99	99.2	0.545
Nonsmoker (%)	91.4	99.5	<0.001
Drinking status (%)			
Nondrinker	68.5	95.4	<0.001
Drink 1–2 times/week	28.2	4.2	<0.001
Drink > 2 times/week	3.2	0.4	<0.001
PA (METs hour/week)	51.1 (50.0, 52.2)	50.4 (49.0, 51.7)	0.409
Sleep duration (7–8 h/day; %)	52.1	58.9	<0.001
Depressive symptoms (%)	11.7	12.1	0.493
Duration of smartphone use (Minutes/day)	321.5 (317.3, 325.8)	363.0 (357.8, 368.3)	<0.001
Physical fitness			
Vital capacity (mL/kg)	24.5 (24.4, 24.6)	18.1 (18.0, 18.1)	<0.001
50 m sprint (Second)	7.38 (7.37, 7.39)	9.11 (9.10, 9.13)	<0.001
1000 m run (Second)	255.1 (254.5, 255.7)		
Pull-up (Times/minute)	3.96 (3.87, 4.05)		
800 m run (Second)		243.0 (242.4, 243.5)	
Sit-up (Times/minute)		32.5 (32.3, 32.7)	

^a^ Obtained using *t*-test for continuous variables and *x*^2^ test for categorical variables. ^b^ BMI: body mass index. PA: physical activity. METs: metabolic equivalents. ^c^ Mean; 95% CI in parentheses (all such values).

**Table 2:** Zero-order correlations between each physical fitness test.

	Males					Females				
	Duration of Smartphone Use	50 m Sprint	1000 m Run	Pull-Up	Vital Capacity	Duration of Smartphone Use	50 m Sprint	800 m Run	Sit-Up	Vital Capacity
Duration of smartphone use	1	0.02	0.02	−0.04 **	−0.01	1	0.00	0.04 **	0.00	0.03
50 m sprint	-	1	0.45 ***	0.33 ***	−0.03 *	-	1	0.43 ***	0.26 ***	−0.07 ***
1000 m run	-	-	1	0.33 ***	0.03 *	-	-	-	-	-
800 m run	-	-	-	-	-	-	-	1	0.26 ***	0.00
Pull-up	-	-	-	1	−0.03 **	-	-	-	-	-
Sit-up	-	-	-	-	-	-	-	-	1	0.07 ***
Vital capacity	-	-	-	-	1	-	-	-	-	1

Results were obtained using correlation analysis and expressed as Pearson’s correlation coefficient (*r*). *: *p* < 0.05; **: *p* < 0.01; ***: *p* < 0.001.

**Table 3:** Association between duration of smartphone use and physical fitness in 6770 males.

	Duration of Smartphone Use			
	Crude Beta	*p* ^a^	Adjusted Beta ^b^	*p* ^a^
50 m sprint	0.02 (−0.01, 0.04) ^c^	0.164	0.03 (0.01, 0.06)	0.004
1000 m run	0.02 (0.00, 0.05)	0.072	0.04 (0.01, 0.06)	0.002
Pull-up	−0.04(−0.06, −0.02)	0.001	−0.03 (−0.06, −0.01)	0.002
Vital capacity	−0.01 (−0.04, 0.01)	0.245	−0.02 (-0.05, 0.00)	0.040

^a^ Calculated by multivariate linear regression analysis. ^b^ Adjusted for grade, race, body mass index, living expenses, physical activity, living status, smoking and drinking habits, depressive symptoms and sleep duration. ^c^ Results are given as betas (95% confidence intervals).

**Table 4:** Association between duration of smartphone use and physical fitness in 4472 females.

	Duration of Smartphone Use			
	Crude Beta	*p* ^a^	Adjusted Beta ^b^	*p* ^a^
50 m sprint	0.00 (−0.03, 0.03) ^c^	0.777	0.01 (−0.02, 0.04)	0.466
800 m run	0.04 (0.01, 0.07)	0.004	0.05 (0.02, 0.08)	<0.001
Sit-up	0.00 (−0.02, 0.04)	0.650	−0.01 (−0.04, 0.02)	0.491
Vital capacity	0.03 (−0.01, 0.05)	0.098	0.00 (−0.03, 0.03)	0.965

^a^ Calculated by multivariate linear regression analysis. ^b^ Adjusted for grade, race, body mass index, living expenses, physical activity, living status, smoking and drinking habits, depressive symptoms and sleep duration. ^c^ Results are given as betas (95% confidence intervals).

## Data Availability

The datasets used and analyzed during the current study are available from the corresponding author on reasonable request.
